# Retrospective investigation of *Echinococcus canadensis* emergence in translocated elk (*Cervus canadensis*) in Tennessee, USA, and examination of canid definitive hosts

**DOI:** 10.1186/s13071-020-04198-9

**Published:** 2020-06-30

**Authors:** BreeAnna Dell, Shelley J. Newman, Kathryn Purple, Brad Miller, Edward Ramsay, Robert Donnell, Richard W. Gerhold

**Affiliations:** 1 Department of Biomedical and Diagnostic Sciences, University of Tennessee College of Veterinary Medicine, 2407 River Drive, Knoxville, TN 37996 USA; 2 Long Island University College of Veterinary Medicine, 720 Northern Boulevard, Brookville, NY 11548 USA; 3 Department of Biology, Lincoln Memorial University, 6965 Cumberland Gap Parkway, Harrogate, TN 37752 USA; 4 Tennessee Wildlife Resources Agency, 3030 Wildlife Way, Morristown, TN 37814 USA; 5 Department of Small Animal Clinical Sciences, University of Tennessee College of Veterinary Medicine, 2407 River Drive, Knoxville, TN 37996 USA

**Keywords:** Cestoda, Coyotes, *Echinococcus*, *Echinococcus canadensis*, Parks, Prevalence, Tennessee, Zoonoses

## Abstract

**Background:**

Few reports of *Echinococcus* spp. have been described in the USA; however, the geographical distribution of *Echinococcus* spp. in wild hosts is increasing consequent to human activities. In the early 2000’s, 253 elk (*Cervus canadensis*) originating from Alberta, Canada were released into the Great Smoky Mountains National Park and North Cumberland Wildlife Management Area in an effort to re-establish their historical range.

**Methods:**

We investigated the prevalence of *Echinococcus* spp. in re-established elk populations in the North Cumberland Wildlife Management Area and the Great Smoky Mountains National Park *via* a retrospective analysis of banked elk tissues and helminth examinations on intestinal contents from coyotes (*Canis latrans*) from the North Cumberland Wildlife Management Area.

**Results:**

Four elk were PCR and sequence positive for *E. canadensis*. Each sequence had 98% or greater coverage and identity to multiple *E. canadensis* genotypes on GenBank. Adult *Echinococcus* spp. were not detected in any of the coyotes examined in this study.

**Conclusions:**

Continued surveillance of this disease in susceptible species in these areas is warranted, and these data further underscore the risk of zoonotic pathogen introduction secondary to wildlife translocation.
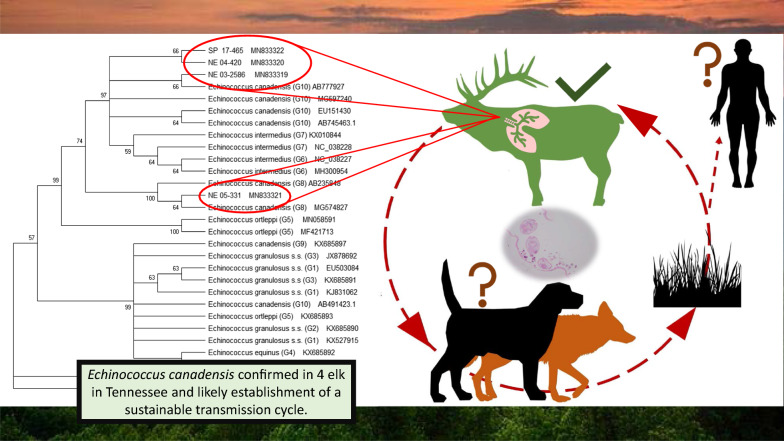

## Background

*Echinococcus* spp. are zoonotic cestode parasites responsible for cystic echinococcosis (CE), one of the designated neglected tropical diseases by the World Health Organization [1]. The parasites cycle between intermediate ungulate hosts and canid definitive hosts as hydatid cysts in various organs and adult worms in the small intestines, respectively. Humans become incidentally infected following ingestion of infective eggs shed in the feces of definitive canid hosts. The resulting pulmonary and hepatic cysts, termed hydatid cysts, are difficult to diagnose and treat in intermediate animal hosts and aberrant human hosts, cause substantial economic loss, and can be fatal as cysts compress host tissues or rupture within the host [2].

There are currently 10 recognized genotypes (G1–G10) which correspond to distinct species within the *Echinococcus granulosus* (*sensu lato*) complex. Each species differs in its host specificity, phenotypic and genetic characteristics, and pathogenicity patterns. The *E. granulosus* (*sensu stricto*) complex (G1–G3) includes the sheep strain, the Tasmanian sheep strain, and the buffalo strain, respectively, and typically involves domestic livestock and domestic canines in its life-cycle. *Echinococcus equinus* (G4) is the horse stain and is specific to equids and *E. ortleppi* (G5) is the cattle strain, and typically cycles between cattle and dogs. *Echinococcus intermedius* (G6–G7), which are grouped with *E. canadensis* under some classification schemes, includes the camel and pig strains. *Echinococcus canadensis* (G8–G10) encompasses the American cervid strain and the Fennoscandian cervid strain, and cycles between cervids including moose, elk, and reindeer and canids. [3–6]. Members of *E. granulosus* (*s.s.*) are most frequently implicated as the causative agents of CE; however, *E. ortleppi* (G5), *E. intermedius* (G6, G7) and *E. canadensis* (G8, G10) are also known contribute to the global burden of human disease [4, 7, 8].

In 2000, the Tennessee Wildlife Resources Agency (TWRA) implemented a re-establishment plan for elk (*Cervus canadensis*) into the Sundquist Wildlife Management and Royal Blue Wildlife Management Area (WMA) public lands in Campbell, Scott, Morgan, Claiborne, and Anderson Counties of Tennessee [9–11]. Royal Blue WMA has since been absorbed into the North Cumberland Wildlife Management Area (NCWMA). Additionally, in 2001, the National Parks Service reintroduced elk into the Cataloochee Valley area of the Great Smoky Mountains National Park (GSMNP). In both locations, elk had been extirpated since the mid-1800s [12]. From 2000 to 2008, a total of 201 elk were released into the NCWMA, and from 2001 to 2002, 52 elk were released into the GSMNP [9, 10, 13]. A 2016 TWRA survey documented 349 elk within NCWMA, suggesting that the reintroduction was successful to date, and populations have remained steady in subsequent years [13]. In both locations, re-introduced elk were originally sourced from Elk Island National Park (EINP) in Alberta, Canada due to the park’s history of disease testing animals and having the Manitoban subspecies (*C. c. manitobensis*), which is considered the closest genetic stock to the extinct eastern elk (*C. c. canadensis*). A portion of the imported elk came from Land Between the Lakes (LBL) National Recreation Area, Kentucky; however, all LBL elk were originally sourced from EINP. Prior to translocation, elk were screened for major pathogens, including brucellosis, bovine tuberculosis, Johne’s disease, anaplasmosis, vesicular stomatitis, bluetongue, epizootic hemorrhagic disease, infectious bovine rhinotracheitis/bovine viral diarrhea, and several strains of leptospirosis [10]. However, antemortem testing for *Echinococcus* was not available. *Echinococcus granulosus* (*s.l.*) is not currently considered endemic in GSMNP or NCWMA, but since the reintroduction of elk, the *E. granulosus* (*s.l*.) strain G10 (i.e. *E. canadensis*) has been presumptively diagnosed in one elk at necropsy. Moreover, an *E. granulosus* (*s.l*.) infection has been suspected in several other elk [14]. No previous reports of echinococcosis in wildlife in this region exist, although they are well documented in wildlife in Canada [15, 16].

With the reintroduction of elk into the NCWMA and GSMNP ecosystems, a pathway for the maturation and spread of *Echinococcus* spp. was newly recreated. It is an emerging concern that the transmission of *Echinococcus* spp. from the translocated animals into wild or domestic canine populations and other sympatric cervids has occurred, thereby establishing a sustainable transmission cycle and reservoir for the disease. This creates a public health risk, as the GSMNP hosted 12.5 million recreational visitors in 2019 [17]. Similarly, NCWMA is a multi-purpose public land that hosts large numbers of visitors and issues 15 elk harvest permits annually [18]. Due to the high tourist load in these recreational areas and the presence of wild canids (coyotes, foxes) and free-roaming domestic dogs, both of which can serve as definitive hosts, there is increased opportunity for wildlife and domestic animal contact, as well as zoonotic transmission [19].

This study describes *E. granulosus* (*s.l*.) lesions and molecular characterization from necropsied elk from NCWMA and GSMNP and investigates parasite transmission in the NCWMA by examining coyote intestinal samples for eggs or protoscoleces. The establishment of a baseline prevalence and ecology data of this pathogen will help fill a critical void in the current awareness of the parasite. Due to the zoonotic potential of this pathogen, this information is vital to informing wildlife management policy, clinical medical and veterinary medical practice, and public health efforts [20].

## Methods

A retrospective search of the University of Tennessee College of Veterinary Medicine (Knoxville, Tennessee) pathology archive spanning 17 years (2000–2017) was conducted to find all necropsy cases of suspected *E. granulosus* (*s.l.*) in elk. Archived histology slides of all selected cases were reviewed by a board-certified pathologist (S. J. Newman) to confirm the presence of *E. granulosus* (*s.l.*) organisms or characteristic hydatid cysts and brood capsules within archived tissue.

**Fig. 1:**
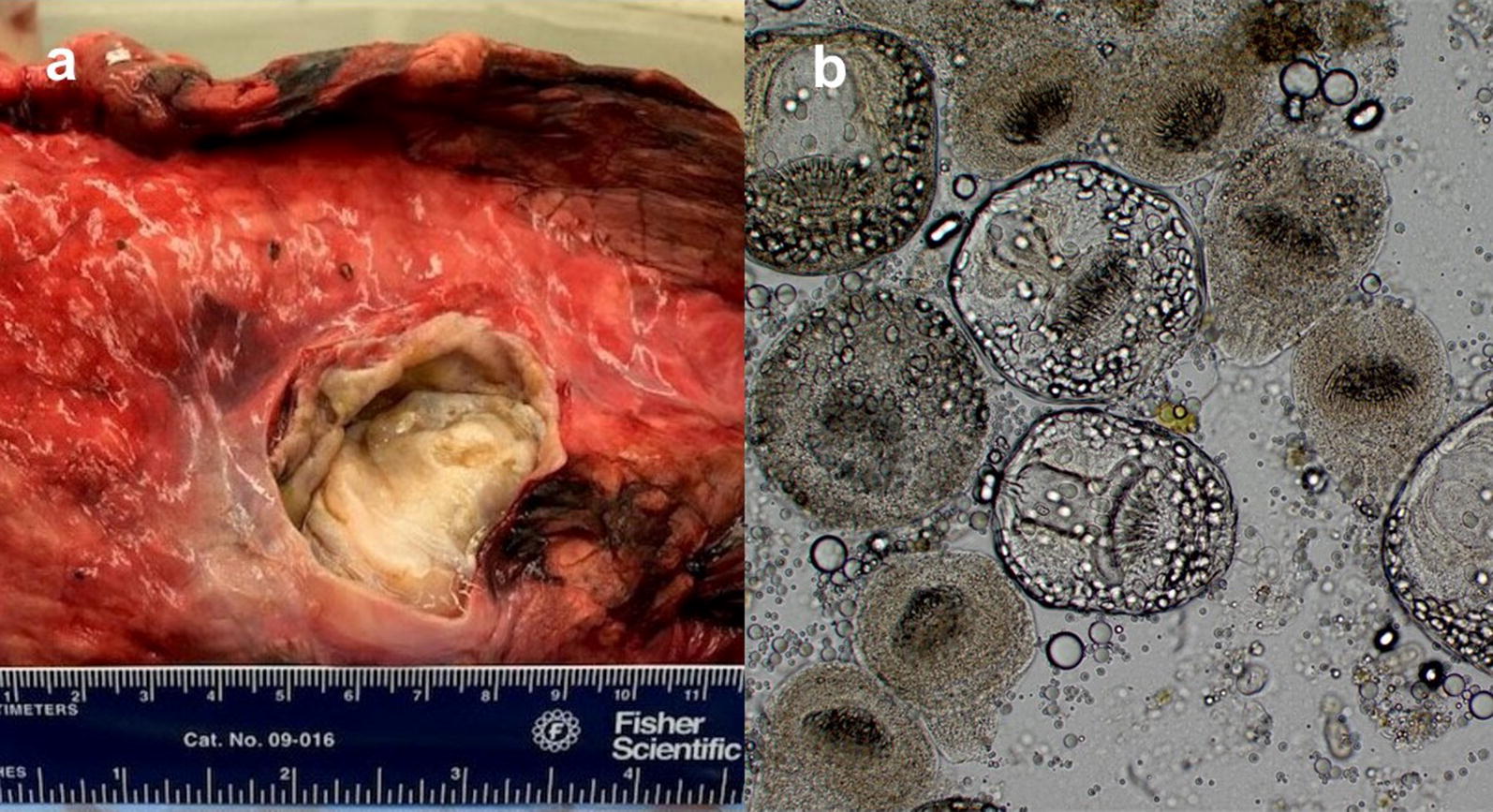
**a** Photograph of a hydatid cyst within the lung tissue of elk SP 17-465 at gross necropsy at the University of Tennessee, 2017. Ruler with inches and centimeters for scale included in photograph. **b** Photomicrograph of invaginated protoscoleces isolated from within aspirate taken from a hydatid cyst of elk SP 17-465 at gross necropsy. Image provided by Heidi Wyrosdick

Tissue samples were cut from paraffin blocks from all identified cases with lesions consistent with *E. granulosus* (*s.l.*) for DNA extraction and subsequent PCR testing to confirm presence of *E. granulosus* (*s.l.*). An additional histology slide was cut after the 10 µm tissue PCR slices and then stained to determine if organisms had been uncovered at the depth of the corresponding PCR sample. Separate microtome blades were used for each block, and microtomes were cleaned thoroughly with DNA AWAY (Thermo Fisher Scientific, Waltham, MA, USA) between blocks. Extraction of DNA was performed using Qiagen DNeasy Blood & Tissue® extraction kit, according to manufacturer instructions. PCR was completed using *cox*1 primers targeting the parasite mitochondrial cytochrome *c* oxidase subunit 1 gene with sequences as follows: COI-F: 5′-TTT TTT GGG CAT CCT GAG GTT TAT-3′ and COI-R: 5′-TAA AGA AAG AAC ATA ATG AAA ATG-3′ [21, 22]. Cycling conditions for PCR were performed in an automatic thermocycler were as follows: after an initial denaturation for 1 min at 95 °C there were 40 cycles consisting of 1 min at 95 °C, 1 min at 50 °C, and 1 min at 72 °C, with a final extension step for 10 min at 72 °C. Both DNA extraction and PCR negative controls were used in PCR reactions to detect contamination. The PCR products were examined using gel electrophoresis in 1.5% agarose gel. Bidirectional sequencing of amplicons was performed at the University of Tennessee sequencing facility (Knoxville, TN, USA). The obtained sequences were compared in GenBank using Basic Local Alignment Search Tool (BLAST). One sample was obtained from the pluck of a freshly killed elk on the same day it was admitted to the University of Tennessee necropsy service (SP 17-465; Fig. 1a). For this specimen, hydatid cysts were observed grossly in the elk lung. Tissue from the cyst wall and fluid from within the cyst were sampled with a sterile scalpel and syringe, respectively, and used for the PCR reaction as described above. In addition, the fluid from the cyst was examined by light microscopy for characteristic findings of *Echinococcus* spp. protoscoleces (Fig. 1b).

Coyote carcasses from within NCWMA were provided by TWRA for examination. Restricted necropsies limited to the gastrointestinal tract were performed. Fecal samples were collected directly from the large intestine of the animals. Fecal flotations using Sheather’s sugar solution with a water step were performed on ~1 g of feces to identify any helminth eggs and coccidian-type oocysts. The gastrointestinal tract from the pylorus of the stomach to the cecum was removed and sieved using Grainger mesh sieves down to the 400 µm mesh. Sieved intestinal contents were preserved in 70% ethanol and examined under a dissecting scope to morphologically identify helminths. Any taeniid eggs or protoscoleces were subject to PCR using *cox*1 gene for molecular identification [21, 23].

## Results

**Fig. 2:**
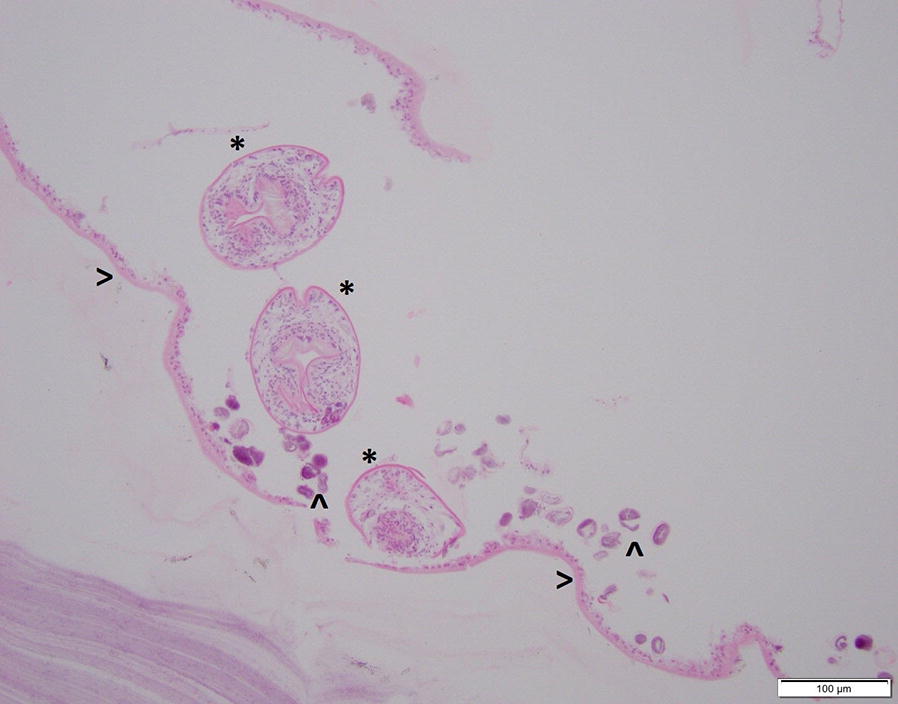
Histological section of a hydatid cyst from elk 07-1. The brood capsule (>) containing three characteristic protoscoleces (*) and mineralized concretions (calcareous corpuscles) (^) can be seen

Of 103 elk necropsy records examined, 14 (13.6%) reports matched selected search criteria based on gross examination. Of these, 7 of the 14 cases (50%) that were examined by the pathologist showed histological findings consistent with or suggestive of *Echinococcus* infection (Fig. 2). The other 7 cases were excluded from further study based on a lack of histological evidence of *Echinococcus* infection. Of the 7 archived necropsy cases, only 4 cases demonstrated identifiable brood capsules or protoscoleces. All 7 cases showed evidence of non-specific cyst wall present within lung tissue. Cause of death was not attributed to *Echinococcus* infection in any of the seven cases.

**Fig. 3:**
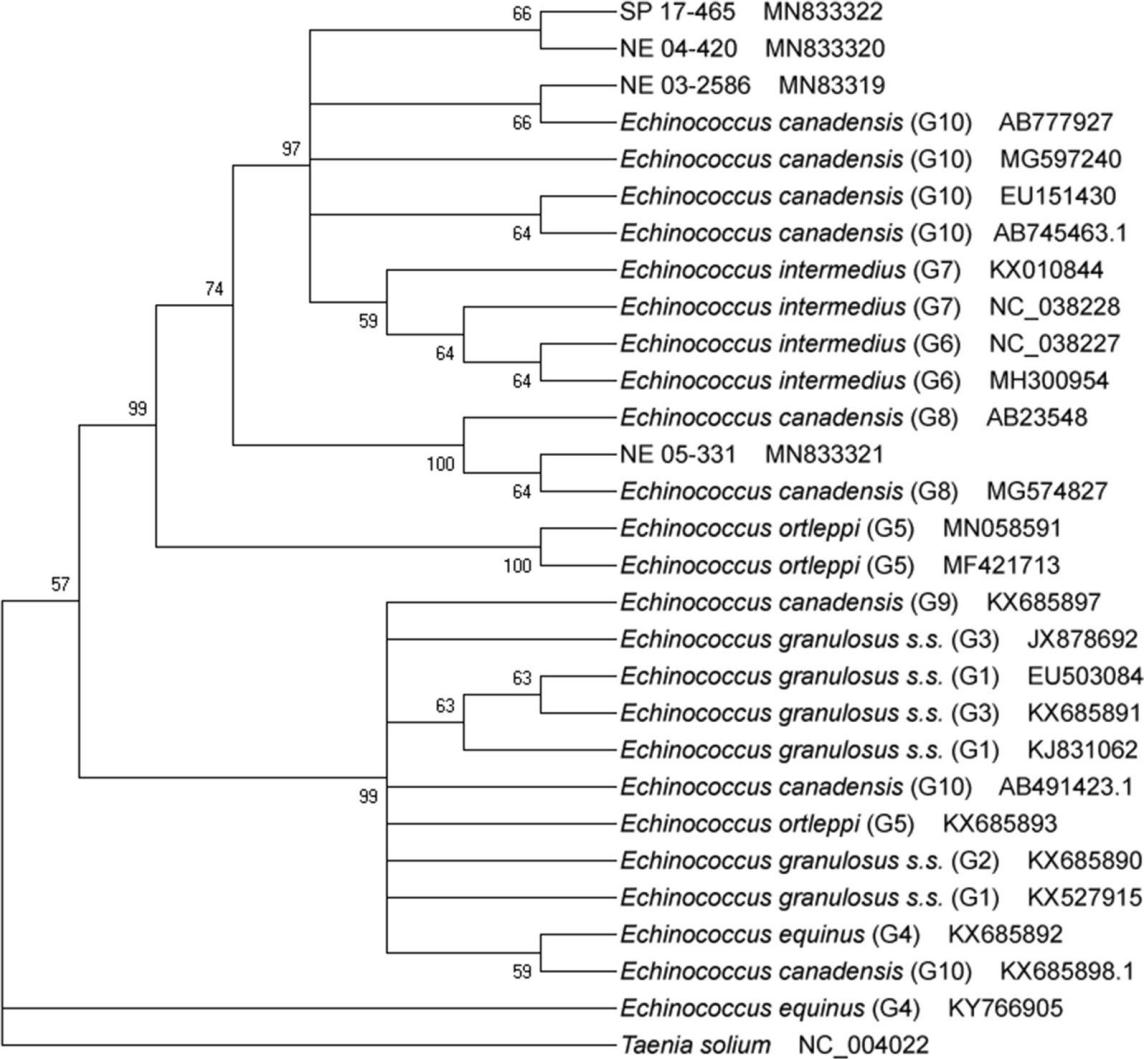
Evolutionary relationship of four *Echinococcus canadensis* isolates from elk (NE 04-420, NE 05-331, NE 03-2586, and SP 17-465) based on *cox*1 sequences. Evolutionary history was inferred by the Neighbor-Joining method using the program MEGA. Percentage of replicate trees in which associated taxa cluster together > 50% of times in the bootstrap test displayed at nodes (1000 replications). *Taenia solium* serves as the outgroup

Three of the seven (42.9%) paraffin-embedded tissue sections were PCR positive using the *cox*1 gene target (Table 1). The single sample obtained from elk SP 17-465 at necropsy was PCR-positive. Of these four PCR positive samples, three had histological evidence of *E. granulosus* (*s.l.*) parasites. Two of the four archived cases with histological evidence of infection were PCR-negative. Sequence analysis of the four consensus sequences *via* NCBI Genbank disclosed at least 98% coverage and 98% identity to multiple *E. canadensis* genotypes. Nucleotide sequences were submitted to NCBI Genbank for each of our four samples. Accession numbers and BLAST result metadata are described in Table 1. Phylogenetic alignment of the *cox*1 sequences resulted in a 324-bp alignment with 305 bp being invariant, resulting in a 94.1% conserved identity among the 4 samples. Elk NE 03-2586 and elk SP 17-465 were the most closely related with a p-distance of 0.0062, while elk 04-420 and elk and 05-331 had the furthest relationship with a p-distance of 0.059. Three of the four samples (SP 17-465, NE 04-420, NE 03-2586) clustered with *E. canadensis* G10 isolates in a phylogenetic tree constructed using the neighbor-joining method. Weak neighbor-joining bootstrap values (47%) support this conservation. Elk NE 05-331 grouped with *E. canadensis* G8 isolates, supported by a strong neighbor-joining bootstrap value of 100% [24, 25]. Phylogenetic relationships among the four *Echinococcus* samples can be seen in Fig. 3.

**Table 1:** Summary of histological presence of protoscolex or brood capsule in lung tissue or liver tissue and PCR results of elk specimens with *Echinococcus* lesions from Tennessee 2002–2017

Specimen ID	Accession year	Histological evidence	PCR	GenBank ID	First BLAST result	Host species	Reference
NE 02-3628	2002	–	–				
NE 03 2586	2003	+	+	MN833319	*E. canadensis* mitochondrion G10 (AB777927.1)	*Alces alces*	Konyaev et al. (2013) [39]
NE 04-420	2004	–	+	MN833320	*E. canadensis* mitochondrion G10 (MG597240.1)	*Bos grunniens*	Wu et al. (2018) [40]
NE 04-800	2004	+	–				
NE 05-331	2005	+	+	MN833321	*E. canadensis* mitochondrion G8 (MG574827.1)	*Canis latrans*	Schurer et al. (2018) [41]
NE 07-1	2007	+	–				
NE 08-46	2008	–	–				
SP 17-465	2017	+	+	MN833322	*E. canadensis* mitochondrion G10 (MG597240.1)	*Bos grunniens*	Wu et al. (2018) [40]

*Note*: Closest match on GenBank for each amplicon from this study are listed. Assigned GenBank accession numbers for submitted sequences are provided

Eleven adult coyotes were necropsied and examined. Adult *E. granulosus* (*s.l.*) parasites were not detected on gross inspection of intestinal content in any of the coyotes included in this study on complete helminthological examination. No Taeniidae-like eggs were identified on fecal floatation from any coyotes included in this study. Sediment of fecal floatation material that was recovered and then centrifuged in water was also PCR-negative for *E. granulosus* (*s.l.*) DNA.

## Discussion

The findings in this study demonstrate a public health concern for potential zoonotic transmission of *Echinococcus granulosus* (*s.l.*) (i.e. *E. canadensis*) for the areas in and surrounding the GSMNP and NCWMA. Introduction of this parasite into a region with no previous documentation of a sylvatic transmission cycle and no public education or prevention strategies creates abundant opportunity for wildlife, domestic animals, and humans to become exposed with little to no recognition of the risks. Furthermore, private agricultural land abuts much of the park, allowing for contact with domestic canids and livestock and the humans that frequent these areas. Concern should be high for the overlap of sylvatic and domestic transmission cycles, as alternative viable intermediate and definitive hosts exist in proximity to reintroduction areas. *Echinococcus granulosus* (*s.l.*) has been previously documented in the southeastern USA in hogs, cattle, and domesticated dogs, although there have previously been no sylvatic cycles documented in the region [6, 26, 27].

The genetic distance between samples in this study suggest that there is some heterogeneity among sequences in Tennessee. For at least three of these samples (SP 17-465, NE 04-420, NE 03-2586) the differences are minor with no genotype differences, which suggests they may be similar or the same strain of *E. canadensis* G10. Elk NE 05-331 exhibited greater genetic distance from other samples and its phylogeny suggested closer relation to *E. canadensis* G8 strains. This may suggest multiple introduction events or introduction of distinct strains of *Echinococcus* in individuals from different geographic sourcing. Further research in translocated elk is warranted to investigate these differences among Tennessee isolates to clarify which strains have been introduced and to establish their origin. Continued surveillance of viable canid hosts for *Echinococcus* may provide insight into which strains are present. Although *cox*1 is a well-established target for looking at interspecies variation, future studies may benefit from multi-locus or whole genome analysis to provide better resolution of *Echinococcus* isolates.

Four of the samples were PCR negative for *Echinococcus*/cestode DNA despite two of these samples having characteristic histological evidence of *Echinococcus* infection. There are several possible explanations for these negative PCR results in the samples with demonstrable protoscoleces and brood capsules, including possible cross-linked DNA secondary to prolonged formalin fixation, which has been previously shown to inhibit DNA amplification [28, 29]. It is also possible that the cestodes were too mineralized and degraded within the cysts to allow DNA extraction, particularly if there was a protracted latency between the death of the animals and the submission to necropsy. Alternatively, samples taken from the archived paraffin blocks did not capture sections of cyst or parasite DNA.

No canids included in this investigation were positive for taeniid eggs or protoscoleces on intestinal or fecal examination or PCR from intestinal content for *Echinococcus* spp. Positive canids would support the hypothesis of sustained *Echinococcus* transmission in the reintroduction areas in addition to being present in elk imported from Canada. Coyotes were opportunistically sampled by TWRA from areas adjacent to and within the elk’s range. All coyotes necropsied were either killed on private property or found dead. Our sample size for surveillance of definitive canid hosts was small and only included coyotes. Future surveillance should include other canids active in both areas, including red foxes (*Vulpes vulpes*), gray foxes (*Urocyon cinereoargenteus*) and potentially domesticated dogs. There are no thriving populations of red wolves (*Canis rufus*) in the GSMNP, following a failed reintroduction of red wolves [30]. Although the canid sample size was small in this study, if the negative fecal results are truly representative of the canid population, the lack of a large canid predator in the GSMNP may be protective against the establishment of an efficient transmission cycle. However, further intensive canid helminth research in the areas is needed to determine if this association is accurate. An active sampling strategy and recruitment of multiple stakeholders (e.g. landowners, resource agencies, wildlife biologists, etc.) to provide specimens may prove useful in the future to more concretely rule out the establishment of an ongoing transmission cycle. In future studies, PCR on fecal homogenate, even in the absence of taeniid eggs on floatation, may be considered as an adjunct diagnostic tool [31].

Three of the four *Echinococcus* positive elk (NE 03-2586, NE 04-420 and NE 05-331) were confirmed to have been part of the stock imported to the region by ear tag number. We suspect that one of the *Echinococcus* positive elk (SP 17-465) was the offspring of one of the originally translocated elk, but we were unable to definitively confirm this. This individual was potentially born in Tennessee, as the last elk was imported to the region in 2008. This suspicion warrants further examination of various intermediate and definitive hosts for this parasite in the region. If this elk were to be a confirmed offspring, this would provide compelling evidence for the establishment of a sylvatic transmission cycle in an area with no previous documentation of the disease, even in the absence of *Echinococcus* positive canid definitive hosts in this study, as this parasite is not vertically transmitted.

## Conclusions

Wildlife translocations have remained a popular and often successful conservation tool to re-establish or augment declining or extirpated populations; however, relatively little emphasis has been placed on disease risk until recently. This neglect is in spite of many documented cases of introduction of novel diseases secondary to translocation efforts, such as with parvoviral enteritis in raccoons (*Procyon lotor*) in West Virginia, rabies from translocated raccoons to local skunks (*Mephitis mephitis*) in West Virginia, brucellosis and tuberculosis in translocated plains bison (*Bison bison*) in Montana, and *Echinococcus multilocularis* in European beavers (*Castor fiber*) in the UK [30–37]. Furthermore, translocation of animals inherently includes numerous stressors, including transport, handling, capture, confinement, diagnostic screening, and release into unfamiliar environments; it is well documented that increases in these stressors are associated with diminished immune function [38]. Potential alterations in immune function during the translocation process may increase the opportunity for infectious diseases to establish in the hosts and allow the introduction of novel pathogens into immunologically naïve populations with potentially serious consequences to the native wildlife, domestic animals and humans. The findings of this study underscore the need for thoughtful, evidence-based best practices in weighing the benefits of reintroduction efforts against the risk of novel pathogen introduction, and a robust process to identify and appropriately mitigate potential disease risks in the translocation of wildlife species.

## Data Availability

The datasets used and/or analyzed during the present study are available from the corresponding author on reasonable request. The newly generated nucleotide sequences were submitted to the GenBank database under the accession numbers MN833319-MN833322.

## Abbreviations

NCWMA: North Cumberland Wildlife Management Area
GSMNP: The Great Smoky Mountains National Park
CE: Cystic echinococcosis
TWRA: Tennessee Wildlife Resources Agency
WMA: Wildlife Management Area
EINP: Elk Island National Park
LBL: Land Between the lakes
